# 3. Transient acute coronary syndrome: delayed reaction to infliximab in an adolescent with juvenile idiopathic arthritis

**DOI:** 10.1093/rap/rky031.002

**Published:** 2018-09-20

**Authors:** Katerina M Achilleos, Peter Bale, Aravind Shastri, Narman Puvanachandra, Kate Armon

**Affiliations:** 1 Rheumatology, Norfolk and Norwich University Hospital, Norwich, UNITED KINGDOM; 2 Paediatric Rheumatology, Norfolk and Norwich University Hospital, Norwich, UNITED KINGDOM; 3 Paediatric, Norfolk and Norwich University Hospital, Norwich, UNITED KINGDOM; 4 Paediatric Ophthalmology, Norfolk and Norwich University Hospital, Norwich, UNITED KINGDOM

**Introduction:** We report a case of a 15 year old girl with extended oligoarticular juvenile idiopathic arthritis (JIA) and chronic bilateral uveitis who had undergone a delayed adverse event to the infliximab biosimilar remsima, resulting in a tachyarrhythmia, significantly elevated troponin I and a diagnosis of transient acute coronary syndrome. This is the first reported case in the paediatric and adolescent population.

**Case description:** Diagnosed at the age of 15 months with oligoarticular juvenile idiopathic arthritis, rapidly transforming to the extended subtype, her arthritis was eventually well controlled with methotrexate and NSAIDS. ANA was positive. On routine ophthalmological assessment asymptomatic uveitis was noticed. Over the years this has become bilateral, chronic and difficult to control despite regular courses of glucocorticoid eye drops and oral preparations. After secondary failure to adalimumab, infliximab 6mg/kg monthly was commenced in 2016, predominantly to achieve control of her uveitis. Initial response was promising and both eyes and joints remained quiet for 10 months. However, following cessation of oral steroids and reduction of steroidal eye drops, with remsima extended to six-weekly infusions, there was serious reactivation of bilateral uveitis. Four doses of remsima were administered fortnightly, followed by the monthly regimen. Topical and oral steroids were restarted. During a couple of these infusions, in early 2018, brief episodes of flushing, dyspnoea and chest tightness ensued but rapidly resolved on slowing down the infusion rate to six-hourly. Hydrocortisone and chlorphenamine were co-administered. Twenty- four hours following her last remsima infusion in May 2018, palpitations, followed by chest pains developed. This was associated with feeling clammy, sweating and breathless. Symptoms persisted for eight hours, which prompted an emergency admission. Heart rate was recorded as 203 beats per minutes. Blood pressure 108/56mmHg. Palpitations ceased just prior to obtaining an ECG and unfortunately the rhythm was not captured. ECG post-termination of palpitations was of normal sinus rhythm without ischaemic changes. Blood tests demonstrated a significantly elevated troponin I a t 1827ng/L (normal range <15.7ng/L). D-dimer 8422ug/L, CRP 4, lactate 0.9 and CK 153u/L. Electrolytes, bone profile, lipid profile, eosinophils and remaining blood tests were unremarkable. Viral screen was negative. Infliximab drug level was <0.3ug/mL and anti-infliximab antibodies <10ng/mL. There was no personal or family history of cardiac disease or arrhythmias. Differentials at this stage included a delayed drug reaction resulting in a tachyarrhythmia, myocarditis or a thromboembolic event. On further discussion with Great Ormond Street Hospital, the advice was to perform a CT pulmonary angiography. CT coronary angiogram and echocardiogram did not yield any sinister pathology. During the observation period, no further arrhythmias arose. Follow-up was arranged with a 48 hour tape. Repeat troponin had normalised within seven days. Remsima has since been discontinued and the patient is currently undergoing a washout period of 12 weeks before proceeding to the next line biologic, tocilizumab.

**Discussion:** Subjects who develop antibodies to infliximab are thought to be at a three-fold increased risk of developing infusion reactions. Side effects at the time of the infusion including: flushing, chest tightness, palpitation, dyspnoea, hypotension and even cardiopulmonary reactions have been documented by the manufacturers, however troponin rise and arrythmias in children are unheard of. For many, resolution of symptoms occurs on slowing down the infusion rate. Arrythmias and palpitations have been reported during infliximab and biosimilar infusions in the adult population in the region of 4-6%, however delayed cardiac reactions are less common and most adult patients are expected to have some form of underlying cardiovascular disease. There have only been three case reports of infliximab related acute coronary syndrome in adults. To our knowledge, this is the first documented case in the adolescent and paediatric cohort. Of the three case reports in the literature, one had cardiovascular risk factors, whilst the other two were both middle aged males, who had developed an acute coronary syndrome during the fifth infusion and three days after the infliximab infusion respectively. The latter had received long term non-steroidal anti-inflammatory drugs and corticosteroids. All three had ischaemic ECG changes. Despite the inability to capture the rhythm prior to its self- termination, a documented reading of a heart rate >200 beats per minute was strongly suggestive of a tachyarrhythmia, which would explain the symptoms experience by the patient. A significantly elevated troponin however, could not be explained by a tachyarrhythmia alone. One hypothesis for developing an acute coronary syndrome, is the vasodilatation role of TNF in the maintenance of myocardial vascular perfusion through the induction of nitric oxide. It is also capable of inhibiting apoptosis of myocardiocytes and attenuation of cardiac stimulation by the sympathetic nervous system through β-receptors. The administration of Infliximab, which is a potent anti-TNF antibody can neutralise both soluble and membrane-bound TNF which can suspend these homeostatic mechanisms, resulting in deprivation of first line defences and leading to coronary vasoconstriction and hypoperfusion. Why patients without prior cardiovascular disease develop such symptoms is still unclear and may be a further scope for research.

**Key Learning Points**: Potent anti-TNF agents have the theoretical ability of causing coronary vasoconstriction and hypoperfusion. Albeit rare, patients presenting with chest pain, during or after the infusion should have the appropriate coronary biochemistry and investigations performed. Young people with no risk of cardiovascular disease can develop transient acute coronary syndrome in response to potent anti-TNF agents. Doubling the frequency of infusion in attempt to regain control of disease after secondary failure should be done with caution and may increase the risk.

**Disclosure: K.M. Achilleos:** None. **P. Bale:** None. **A. Shastri:** None. **N. Puvanachandra:** None. **K. Armon:** None.

